# 

**Published:** 1873-12

**Authors:** 


					Bibliographial. 383
An Investigation Concerning the Mechonism of the Ossicles of
Hearing and the Membrane of the Round Window. By Charles
H. Burnett, M. D., Fellow College of Physicians, Philadelphia.
From the Archives of Ophthalmology and Otology. William
Wood & Co., Publishers, New York.
TO READERS AND CORRESPONDENTS.
? 1
Communications intended for publication, and exchange journals and paper
should be addressed to the Editor of. the American Journal of Denta
Science, 259 N. Eutaw St., Baltimore, Md. All letters relating to the busine
of the journal, or containing cash remittances, to be directedto Messrs. Snowds
& Cowman. Articles for publication should be received by the editor at lea
three weeks previous to the first day of the month on which the number in whi
it is designed for them to appear, is issued.
THE
AMERICAN JOURNAL
OF
DENTAL SCIENCE.
F. J. S. GORGAS, M. D.,D.D. S., Editor,
The Journal is issued on the first of each month and contains not less
han fifty pages of reading matter in each number, exclusive of adver-
isements, making a volume of six hundred pages per annum.
In each number will be found Original Communications, Corres-
>ondence, Selected Articles, Monthly Summary, Bibliographical No-
ices and Editorial Matter, and every effort will be exerted to render
he Journal worthy of the patronage of the Dental profession.
A generous co-operation is respectfully solicited from the members
f the profession ; and as the Journal has now a printing office of its
>wn which materially lessens the cost of its publication, it will be
ssued at the reduced subscription price of
$2.30 Per Anamm in Advance.
Communications are respectfully solicited on all subjects pertain-
ng to the practice of dentistry, as well as reports of cases occurring
in dental practice
RATES OF ADVERTISING.
1 Page.
h ?"
i "
1 ?
1 MO.
$15 00
10 00
7 00
5 00
2 MO.
$22 50
15 00
11 00
8 00
3 MO.
$30 00
20 00
15 00
11 00
6 MO.
$50 00
30 00
20 00
15 00
12 MO.
$100 00
50 00
30 00
20 00
All original communications designed for publication, works for
review, new instruments for notice, exchanges, &c., should be directed
to the editor, 259 N. Eutaw St., Baltimore, Md.
All advertisements and subscriptions should bei addressed to
SNOWDEN & COWMAN,
Publishers of the Am. Journal of Dental Science. '
86 W. Fayette St. Bet. Charles & Liberty Sts.,
BALTIMORE, Md.

				

